# Next generation of heart regenerative therapies: progress and promise of cardiac tissue engineering

**DOI:** 10.1038/s41536-021-00140-4

**Published:** 2021-06-01

**Authors:** Miguel F. Tenreiro, Ana F. Louro, Paula M. Alves, Margarida Serra

**Affiliations:** 1 iBET, Instituto de Biologia Experimental e Tecnológica, Apartado 12, 2781-901 Oeiras, Portugal; 2 Instituto de Tecnologia Química e Biologica António Xavier, Universidade Nova de Lisboa, Av. da República, 2780-157 Oeiras, Portugal

**Keywords:** Tissue engineering, Induced pluripotent stem cells, Stem-cell research, Stem-cell biotechnology, Regenerative medicine

## Abstract

The adult heart is a vital and highly specialized organ of the human body, with limited capability of self-repair and regeneration in case of injury or disease. Engineering biomimetic cardiac tissue to regenerate the heart has been an ambition in the field of tissue engineering, tracing back to the 1990s. Increased understanding of human stem cell biology and advances in process engineering have provided an unlimited source of cells, particularly cardiomyocytes, for the development of functional cardiac muscle, even though pluripotent stem cell-derived cardiomyocytes poorly resemble those of the adult heart. This review outlines key biology-inspired strategies reported to improve cardiomyocyte maturation features and current biofabrication approaches developed to engineer clinically relevant cardiac tissues. It also highlights the potential use of this technology in drug discovery science and disease modeling as well as the current efforts to translate it into effective therapies that improve heart function and promote regeneration.

## Introduction

Cardiovascular diseases (CVDs) have been recognized as a major concern for global health among noncommunicable diseases^1^, and despite half-century of advances in cardiovascular science and preventive medicine, CVDs continue to yield high mortality and morbidity rates worldwide^2^. Revascularization interventions or pharmacological treatment allow in many cases to salvage the heart and prevent transplantation, even though such strategies are not recognized as a definitive cure^3^.

The adult human heart has a limited regenerative capacity^4^ and so regenerative medicine therapies have shed new hopes to repair or replace damaged hearts. The marginal benefits reported by cell-based^5–8^ and cell-free^9–11^ approaches have grossly been associated with inappropriate delivery and retention, as well as ineffective therapeutic efficacy^12^. In the past decades, tremendous advances have been witnessed in the field of tissue engineering (TE), especially concerning stem cell engineering, the development of functional biomaterials and biomimetic scaffolds, and the implementation of biofabrication tools for the generation of complex biological structures with high resolution^13^. This progress has set a solid background to rethink current therapeutic approaches and design new bioengineering therapies^14^. Nonetheless, creating fully matured and functional cardiac tissue in vitro is still challenging. Despite this, it is becoming possible to robustly engineer miniaturized tissue versions able to compete, if not replace, commonly used platforms to assess drug safety and model human physiopathology to guide efficient drug discovery^15^.

This review highlights recent progress in cardiac stem cell bioengineering, with a particular focus on the identification of essential requirements for the design of clinically relevant human cardiac tissues: (i) the cell sources, (ii) bioinspired strategies to generate cardiomyocytes (CMs) with improved maturity, and (iii) biofabrication methodologies. The potential use of this technology in drug discovery science and disease modeling as well as the current efforts to translate it into effective heart regeneration therapies is also discussed.

## Cell sources to engineer cardiac tissue

Cardiac muscle is the functional unit of the heart, as it is involved in force generation and propagation of electrical signals through the CM-rich tissue to allow rhythmic pump contraction. Human cardiac muscle is composed of various cell types, the most abundant being CMs, pericytes, endothelial cells, and fibroblasts. The proportion of each cell population in the heart is still a matter of discussion, but there are evidences that CMs occupy the majority of the heart volume (roughly 75%) and account for 30–40% of all cells^16^. State-of-the-art analyses of single-cell/nuclei RNA-sequencing transcriptomic data of six heart regions across several unrelated individuals have clarified that CMs are the most prevalent cardiac cell population in the heart, while also highlighting a surprising cellular heterogeneity among CM, pericyte, endothelial, and fibroblast cell populations^17^. It appears that the cellular composition of cardiac muscle and its transcriptional signature varies across the heart in order to ensure that each anatomical region is highly specialized in a given function. Such complexity undeniably elevates the challenge of engineering artificial cardiac muscle.

Typically, engineering a contractile cardiac muscle requires foremost a source of functional CM. The first engineered cardiac tissue models relied on nonhuman cell cultures of different species, including cell lines of rat embryonic myoblasts (H9c2^18^) and mouse CMs (HL-1^19^) as well as primary CMs isolated from chicken embryos^20^ and neonatal rats^21^. Despite limitations regarding human tissue availability, human adult CMs can be isolated from myocardial biopsies^22^; however, soon after isolation, CMs undergo a profound structural and functional remodeling, leading to cell dedifferentiation and loss of viability^23^. Recently, applying near-physiologic electromechanical stimuli during 24 h proved capable of inactivating CM remodeling after isolation^24^. Long-term culture (up to 4 months) of adult human myocardial muscle strips subjected to biomimetic stimuli similarly prevented pronounced CM remodeling, even though changes at the structural, mechanical, and gene expression levels were detected^25^. Immortalized adult human ventricular CM cell lines have also been established by the fusion of primary cells with SV40-transformed human fibroblasts to create cardiac models^26^, but their poor recapitulation of human physiology discourages its use.

Due to their high self-renewal capacity and ability to generate any cell type of the human body, pluripotent stem cells (PSCs) are becoming an attractive alternative for cardiac TE. These PSCs can either be derived from the inner cell mass of the blastocyst, named embryonic stem cells (ESCs)^27^, or by forced expression of pluripotency genes through the delivery of the “reprogramming factors” *Oct3/4*, *Sox2*, *Klf4*, and *c-Myc* to somatic cells, termed induced PSCs (iPSCs)^28,29^. In particular, human iPSC (hiPSC) derivatives better capture patient-specific physiology^30,31^, besides surpassing the ethical concerns associated with human ESC (hESC). Acquired knowledge on stem cell biology together with the advances in PSC technology has allowed the establishment of robust differentiation protocols capable of obtaining every subtype of CM (ventricular, atrial, and pacemaker) with a high degree of purity^32^. Briefly, temporal modulation of the Wnt/β-catenin signaling pathway is usually performed by first promoting activation for mesoderm formation and subsequent inhibition for cardiac specification^33,34^. The use of small molecules (e.g., CHIR99021, IWP2, IWR1) to control the Wnt/β-catenin signaling pathway is currently preferred to growth factors/recombinant proteins due to their better diffusion properties and moderate cost^32,35,36^.

**Fig. 1:**
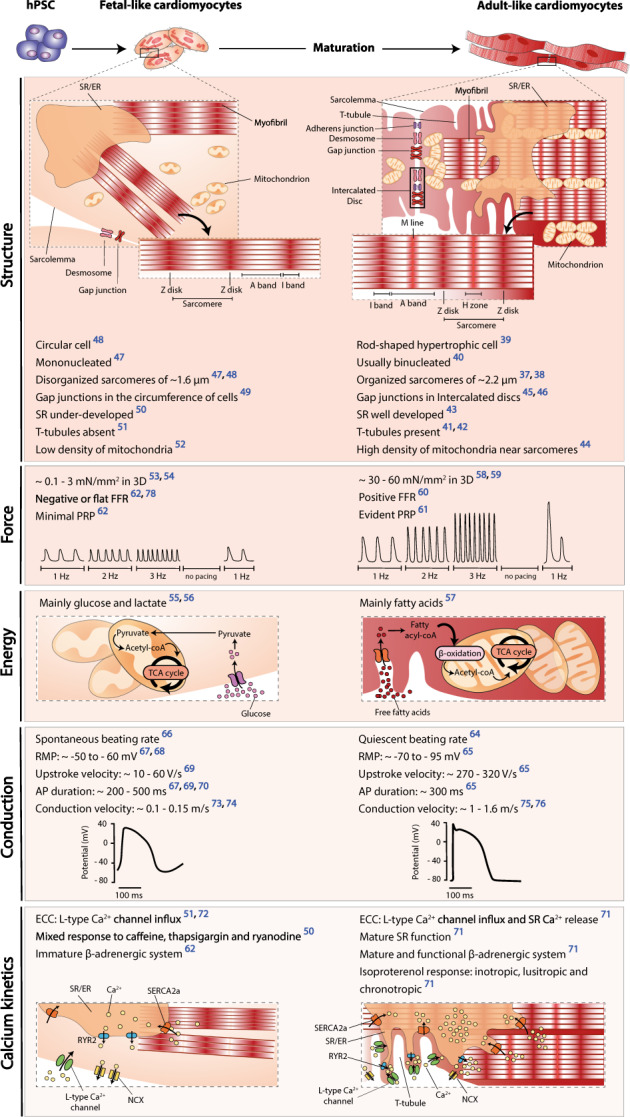
Differences between human pluripotent stem cell-derived cardiomyocytes (hPSC-CMs) and ventricular cardiomyocytes from adult cardiac tissue. hPSC-CMs obtained from the differentiation of pluripotent stem cells present fetal-like features in respect to structural and ultrastructural organization, contractile force, metabolism, and electrophysiological function. AP action potential, ECC excitation–contraction coupling, FFR force–frequency relationship, hPSCs human pluripotent stem cells, NCX sodium–calcium exchanger, PRP post-rest potentiation, RMP resting membrane potential, RYR2 ryanodine receptor type 2, SERCA2a sarcoplasmic/endoplasmic reticulum calcium-ATPase 2a, SR/ER sarcoplasmic/endoplasmic reticulum.

A lot of effort has been put to evaluate the maturity state of human PSC-derived CMs (hPSC-CMs) and it is unanimous that, after differentiation, these cells are more similar to fetal rather than adult CMs (Fig. 1). Adult ventricular CMs display a remarkably organized structure with sophisticated intracellular organelles optimized for contraction. Among these components are densely packed aligned sarcomeres^37,38^ within a hypertrophic^39^ (usually) binucleated^40^ cell; the sarcolemma and a vast network of transverse tubules (T-tubules)^41,42^ along the Z-lines to allow for fast depolarization; a functional longitudinal sarcoplasmic or endoplasmic reticulum (SR/ER) and terminal cisternae positioned near T-tubules adapted for quick calcium kinetics^43^; a high density of mitochondria near the contractile apparatus to improve ATP consumption^44^; and intercalated discs with well-developed cell junctions to promote electrical coupling^45,46^. hPSC-CMs have not shown this level of cellular complexity^47–52^, as they are less fit for contraction^53,54^ and are incapable of matching the metabolic demands of adult CMs^55–57^ needed for significant force generation^58–61^, which is apparent in their negative force–frequency relationship (FFR)^62,63^ and minimal post-rest potentiation (PRP)^62^. Due to a dissimilar ion-channel density, hPSC-CMs exhibit a distinct action potential (AP) profile from adult CMs^64,65^, resulting in asynchronous and spontaneous beating rate^66^, less negative resting membrane potential (RMP)^67,68^, slower upstroke velocity^69^, and irregular AP duration^67,69,70^. In fact, the immature ultrastructure of hPSC-CMs not only affects calcium handling^71^ due to an under-developed excitation–contraction coupling (ECC) machinery^50,51,62,72^ but it also slows AP propagation^73,74^ owing to the lack of organized electromechanical junctions compared to adult cardiac tissue^75,76^. Furthermore, these differences are seen at a transcriptomic level, with hPSC-CMs showing preferential expression of fetal sarcomeric gene isoforms (myosin heavy chain: *MYH6* > *MYH7*; troponin I: *TNNI1* > *TNNI3*; titin: *TTN-N2BA* > *TTN-N2B*) as well as lower expression of genes related with electrophysiological function (e.g., *KCNJ2*, *KCND3*, *KCNJ8*, *KCNH7*, *SCN5A*, *HCN4*, *GJA1*, *JPH2*) and ECC (e.g., *CACNA1C*, *RYR2*, *ATP2A2*, *CASQ2*, *SLC8A1*, *PLN*, *ITPR3*, *CAV3*, *BIN1*)^77^.

The maturation defects of hPSC-CMs have gained increased attention over the past decade since their immaturity greatly limits their application in several areas of precision medicine. A better understanding of cardiac development has allowed the identification of relevant players that partake in the transformation of the fetal heart into a competent and efficient organ. Complete maturation of hPSC-CMs has yet to be reported; however, nature-inspired biological strategies and/or engineering solutions proposed over the past few years have undoubtedly made significant contributions to improve the maturation of these cells.

## Bioinspired strategies to improve hPSC-CM maturity

**Fig. 2:**
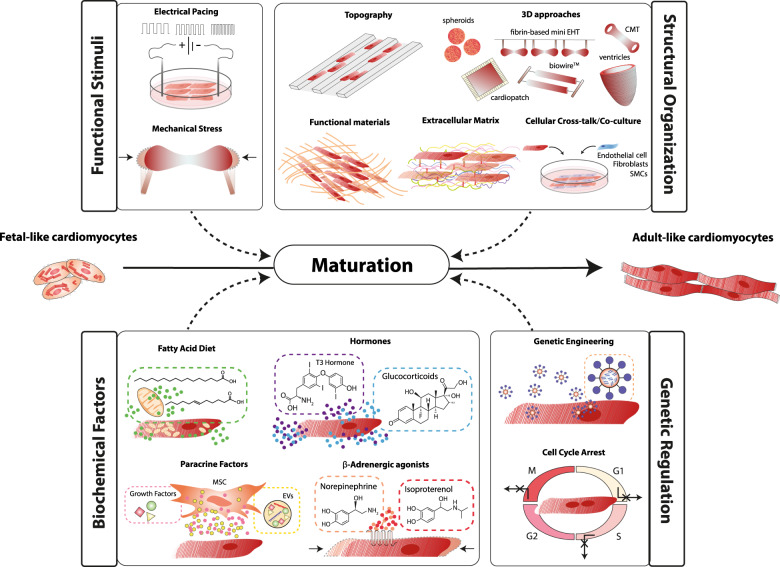
Bioengineering strategies to improve the maturation of cardiomyocytes derived from human pluripotent stem cells (hPSC-CMs). hPSC-CMs can develop adult-like features with training based on functional stimuli, cues to guide their structural organization, relevant biochemical factors, and by manipulating the genetic program. CMT cardiac microtissue, EHT engineered heart tissue, EV extracellular vesicle, MSC mesenchymal stem cell, SMC smooth muscle cell, T3 triiodothyronine.

During cardiac development, several environmental factors including mechanical forces, electrical stimuli, biochemical factors’ gradients, extracellular matrix (ECM) remodeling, and heterotypic interactions drive CM maturation^78^. Maturation is the last phase of cardiac development and corresponds to a series of orchestrated phenotypic events that prepare the fetal heart for efficient and competent life-long pumping function. Some of these events are initiated in utero, such as physiologic hypertrophic growth, sarcomeric isoform switch, cell cycle exit, and a glycolytic-to-oxidative metabolic switch^79^. However, the highly specialized cellular machinery of adult CMs only fully develops years after birth^46^. Even though prolonging time in culture of hPSC-CMs (up to 1 year) improves structural and functional maturation^47,80,81^, these cells still show signs of a fetal phenotype. Early-stage hPSC-CMs transplanted in animal hearts were able to acquire adult-like features^82,83^, providing evidence of their plasticity and ability to be matured if the right environment is ensured. By attempting to mimic nature, various bioengineering approaches have been proposed to promote hPSC-CM maturation (Fig. 2).

### Functional stimuli

In cardiac tissue, CMs are responsive to external electrical stimuli and mechanical forces to perform their function, and these are initiated early during cardiac embryogenesis.

Electrical excitability is a key feature of CMs, dictating electromechanical coupling. The effect of exogenous electrical stimuli on CM maturation was first tested using neonatal rat CMs seeded in collagen sponges and exposed to pulse waves at 1 Hz for up to 8 days, resulting in cell alignment and elongation along the electrical field as well as the formation of intercalated discs with gap junctions expressing connexin-43^84,85^. Both neonatal rat CMs^86,87^ and hPSC-CMs^88^ have also shown a more mature phenotype after being stimulated with pulse frequencies >1 Hz. Interestingly, applying a supraphysiological pacing regimen up to 1 week (increasing pulse frequency from 1 to 6 Hz) to hPSC-CMs seeded in a collagen matrix markedly contributed to cell alignment, establishment of an organized contractile ultrastructure, improved SR/ER function, and electrophysiological profile^89^.

In turn, mechanical stress induces CM rhythmic contraction, which has a direct impact on physiologic hypertrophic growth, cell elongation, tissue alignment, and force generation. By casting neonatal rat CMs in a ring-shaped mixture of collagen and Matrigel^®^ exposed to mechanical stretch, it was demonstrated for the first time the importance of applying stress to induce both ultrastructural and functional maturation of CM^90^. Since cylindrical casting molds provided little tissue deflection, mechanical stimulation via cyclic stretch is now achieved using fibrin-based hydrogels in flexible posts^91^ or in thin elastomeric frames^92^, allowing for auxotonic muscle contraction. These technologies were first established using rodent cells, but were successfully translated to hPSC-CMs^62,93^. hPSC-CMs show alignment along the hydrogel and sarcomeric organization^62^, a metabolic shift towards fatty acid consumption^94^, improved calcium drug sensitivity^95^, and force development^62^, even though the force generated (up to ~1 mN/mm^2^) is still relatively inferior to those of adult cardiac muscle strips (~30–60 mN/mm^2^)^58,59^. More recently, modulation of cyclic^96^ and passive stretching^97^ of hPSC-CMs revealed an impact on tissue functional performance, indicating that there might be ways to further improve tissue force generation. Of note, more complex systems simulating the hemodynamic loads of the cardiac cycle have also been used to improve hPSC-CM maturation^98^.

In addition, CMs can be electrically and mechanically stimulated simultaneously^99–101^, thereby enhancing maturation. In a recent work, hPSC-CMs subjected to combined cyclic stretch in elastomeric posts and a supraphysiological pacing regimen of 2 weeks (increasing pulse frequency from 2 to 6 Hz) resulted in these cells displaying adult-like features never reported before, such as a vast network of T-tubules, inotropic response to isoproterenol, a positive FFR, and noticeable PRP^101^. This represents the most advanced strategy reported for hPSC-CM maturation so far, although these more mature hPSC-CMs still presented inadequate contractile force generation ability (up to ~4 mN/mm^2^) when compared to adult counterparts.

### Structural organization

Cardiac tissue function depends on a highly anisotropic architecture with orderly aligned myofibrils along the direction of muscle contraction, which is impossible to replicate when culturing CMs on standard and simple plastic two-dimensional (2D) surfaces. Promoting this level of cellular organization can be achieved using grooved patterned substrates where hPSC-CMs already showed elongation along the line patterns, electromechanical coupling with neighboring CMs, and electrophysiological improvements^68,102^. Of note, substrate stiffness, groove width, and cell density seem to have an impact on the structural maturation of hPSC-CMs^103^. In fact, the stiffness of a substrate can by itself modulate the mechanical load experienced by hPSC-CMs, affecting the cytoskeleton organization^104^ and contractility^102^. In addition, various natural (collagen^20,89^, fibrin^91^, and Matrigel^®^^90,105^) or synthetic (polyesters^106,107^, polyethylene glycol^108^, polylactones^109^, and elastomers^110^) biomaterials can be used to guide cardiac tissue structural organization, even though their properties (mechanical, topographical, and biocompatibility) influence the degree of maturation obtained. Unfortunately, none of these biomaterials can fully recapitulate the architecture and functional composition of the cardiac ECM. Still, artificial ECM^111^ or decellularized myocardial ECM^112–114^ has been reported to promote hPSC-CM maturation without biomimetic regimens.

Throughout cardiac development, CMs are in direct contact with other cell types. Even though non-myocytes occupy a relatively small volume in cardiac tissue (~25%), they are necessary for CM survival, contractile performance, cell–cell communication, vascular supply, and ECM deposition^115^. Coculture of hPSC-CMs with endothelial cells, either on Matrigel^®^-coated surfaces^116,117^ or Matrigel^®^ hydrogels^118^, resulted in higher expression of proteins related with CM maturity^116^, contributed for ultrastructural organization^116,117^, improved contractility^118^, and calcium handling^116,118^.

Assembling hPSC-CMs into three-dimensional (3D) tissues greatly improves cell–cell communication with more physiological relevance, promoting structural, metabolic, and functional maturation. Engineered tissues can assume various architectures, such as cell sheets^119^, spheroids^120,121^ and organoids^122^, muscle strips in the form of rings^90,123^, ribbons^63,124^ or cables^89,92^, patches^125,126^, and even ventricle analogs^127^. A recent study using a patch platform reported, without exogenous stimulation, advanced hPSC-CM mechanical maturity with a contractile force closer to adult ventricular tissue (~17–22 mN/mm^2^)^126^, even though it had slightly negative FFR and an organized ECC apparatus without pronounced T-tubulation. Nonetheless, 3D approaches are extremely versatile and can be combined with other strategies to enhance maturation and tissue performance (e.g., intensive electrical training^101^, dynamic culture^92^, coculture with other cell types^128–130^). For instance, culturing fibroblasts and hPSC-CMs in muscle strips with a ratio of 1:3 improved cell–cell communication and tissue remodeling^128^, in addition to also contributing to hPSC-CM maturation. Intriguingly, the electrical pacing of hPSC-CM muscle strips in flexible posts requires a supporting network of fibroblasts, otherwise the tissue loses its integrity when electrically stimulated^101,131^. Due to the increasing relevance of 3D maturation-based strategies to the field, we will further detail 3D tissue fabrication methodologies in this review (see “Fabrication of biomimetic cardiac tissues”).

### Biochemical factors

Several soluble and biological factors have been proposed to induce hPSC-CM maturation, such as the type of carbon substrate and hormones. In vivo, CM maturation is characterized by a metabolic shift from glycolysis to preferential fatty acid oxidation, a consequence of cellular hypertrophy and higher energetic demands^56^. hPSC-CMs display metabolic substrate plasticity that can be modulated to promote fatty acid β-oxidation^132,133^. Our group has demonstrated that hPSC-CM structural, functional, and metabolic maturation can be improved in fatty acid-containing medium with galactose to prevent lipotoxicity^134^, when compared with glucose-rich medium. Among hormones, triiodothyronine (T3) is essential during cardiac development^135^, and has revealed to play a key role in inducing hPSC-CM maturation^136^. Furthermore, by combining T3 with dexamethasone, a glucocorticoid hormone, noticeable improvements of SR/ER contribution in ECC and T-tubulation were detected, which otherwise was not seen with each hormone alone^137^.

Biological factors such as those secreted by neighboring cells within the cardiac niche can also positively impact CM maturation state. Recently, hPSC-CM and mesenchymal stem cell (MSC) coculture promoted cellular cross-talk via the release of cytokines and extracellular vesicles capable of modulating hPSC-CM sarcomeric organization, electrophysiology, and metabolism^138^. Another study used a spherical microtissue containing hPSC-CMs as well as cardiac-specific endothelial cells and fibroblasts, and revealed that this tri-cellular interaction upregulated the cAMP/β-adrenergic pathway^130^. An increase in intracellular cAMP levels consequently affected the assembly of connexin-43 gap junctions, besides improving hPSC-CM structural, contractile, metabolic, and electrophysiologic features. Taken together, by identifying key biological factors capable of inducing CM maturation, these studies underline the importance of heterotypic cell interaction in the cardiac microenvironment.

A less conventional approach to induce CM maturation has been the use of β-adrenergic agonists, namely norepinephrine^139^, isoproterenol^140^, and phenylephrine^141,142^. These compounds are capable of causing structural changes and cellular hypertrophy by inducing intensive workload, even though these effects are likely dose-dependent to avoid pathological remodeling and cytotoxicity.

### Genetic regulation

Adult CMs have defined gene regulation mechanisms, ultimately dictating cardiac tissue phenotype and maturation. Overexpressing *KCNJ2* during differentiation resulted in hPSC-CMs with hyperpolarized RMP and eliminated the AP proarrhythmic trait during repolarization, even though calcium handling and structural features were not improved due to early loss of automaticity^143^. In turn, calcium kinetics were significantly improved in hPSC-CMs by forced expression of *CSQ2*^144^. Instead of manipulating the transcriptome, altering the epigenetic state of hPSC-CMs using histone H3 deacetylase inhibitors also showed to promote adult-like expression of calcium handling and conduction genes^145,146^, even though reports regarding electrophysiologic improvements are inconsistent. Priming cells with polyinosinic-polycytidylic acid at the cardiac progenitor stage of differentiation strikingly accelerated structural, electrical, and metabolic maturation of hPSC-CMs, and this acceleration of the developmental clock was associated with increased H3K9ac activation that promoted earlier transcription of cardiac myofilament genes and the Notch ligand *JAG1*^147^. In another study, the inhibition of aberrantly upregulated factors involved in aerobic glycolysis promoted an hPSC-CM metabolic shift towards oxidative phosphorylation, and consequently features of functional maturation^133^. Moreover, the comprehensive analyses of micro-RNA (miR) levels throughout hPSC-CMs’ prolonged culture have identified those that become differentially expressed^148,149^, suggesting alternative routes to modulate gene expression to induce maturation. Let-7 family miRs^150^, miR-1^151,152^, miR-499^152^, and mir-200c^153^, and a combination of miRs (miR-125b, miR-199a, miR-221, and miR-222)^154^ have proved useful to improve hPSC-CM maturation features.

Soon after birth, CMs become nonproliferative cells due to extensive hypertrophic growth and remarkable sarcomere re-organization hindering cytokinesis. Ultimately, these changes result in an increase in binucleation and polyploidy. A recent work revealed that knockout of the ECM protein agrin in neonatal mice resulted in the loss of CM proliferation alongside sarcomere assembly and maturation^155^. As expected, administration of agrin to hPSC-CMs avoided loss of proliferation, but negatively affected gene expression and conduction velocity^155^. By promoting cell cycle cessation, mitomycin C treatment similarly increased sarcomerogenesis in neonatal rat-derived PSC-CMs^156^. Further work will be needed to elucidate how cell cycle arrest can promote hPSC-CM maturation other than structural improvements.

## Fabrication of biomimetic cardiac tissues

**Fig. 3:**
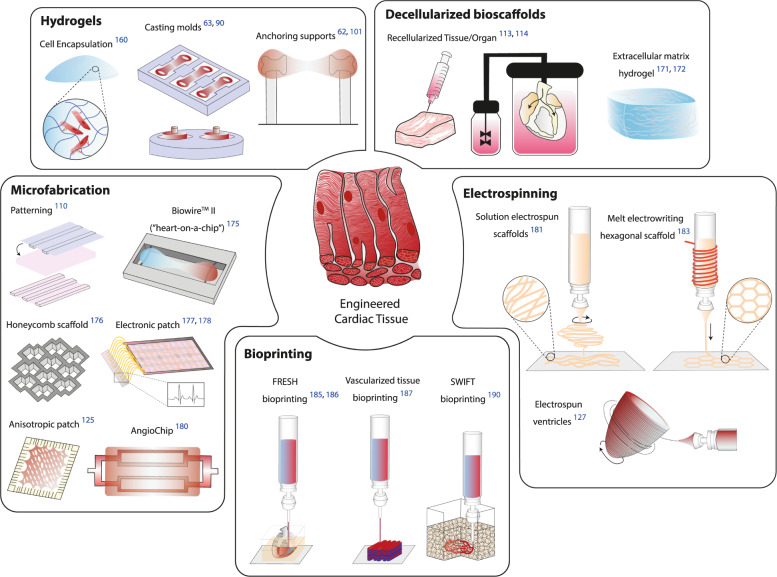
Overview of state-of-the-art biomimetic cardiac tissues and corresponding fabrication approaches. Engineered cardiac tissues range from hydrogels capable of encouraging muscle fiber organization, decellularized scaffolds for tissue/organ bioengineering, microfabricated systems for promoting tissue anisotropy and vascularization, electrospun fibers for close mimicry of tissue architecture, and even printable tissue/miniaturized organs. FRESH Freeform Reversible Embedding of Suspended Hydrogels, SWIFT Sacrificial Writing Into Functional Tissue.

Inducing the maturation of hPSC-CMs is still a challenge in the field of cardiac TE; however, considerable progress has been achieved by seeking innovative approaches. Moving from simple beating cells to physiologically relevant tissues will require further efforts to engineer the complexity of the in vivo conditions. In the following section, we highlight the most promising techniques adopted to date to grow biomimetic cardiac tissue in the laboratory (Fig. 3). A more detailed overview of these current biofabrication methodologies, their advantages and disadvantages for translation to TE and regenerative medicine fields can be found in ref. ^157^.

### Hydrogel method

Hydrogels are the most widely used polymers for cardiac TE due to their biocompatible physical and chemical properties, including high water content, efficient gas and mass transfer (i.e., adequate exchange of oxygen and metabolites), ability to be molded into several geometries, easy to be modified/functionalized in order to include cell-binding sites (e.g., RGD integrin-binding domains and ECM proteins), and scalability^158,159^. Cardiac tissues can be fabricated by joint gelation of cells and a polymer^160^ or by having a casting mold to confine the cells in a given polymeric organization^63,90^. Typically, natural hydrogels^89,91,101^ are preferred for engineering cardiac tissues by promoting cell assembly and subsequent maturation, but functionalized synthetic polymers^106^ or hybrid biomaterials^161,162^ are also suitable options. Cells often self-organized within hydrogels in ways that are different from those found in native tissues, and so it can be advantageous to tailor the hydrogel’s microarchitecture to encourage anisotropic rather than isotropic tissue growth, which can be done by relying on simple methods such as freeze-drying, solvent casting, gas foaming, and unidirectional freezing^163^. Hydrogels are generally soft, with inferior mechanical compliance in comparison to the native heart tissue. Anchoring supports are useful not only to retain hydrogel conformation but also to provide a more adequate mechanical load to CM^62,101^ and facilitate force measurement^87,131,164^. Impressively, the resulting tissues can be several millimeters long (~7–9 mm^62,101^).

### Decellularized bioscaffolds

The main aim of decellularization methodologies is to generate scaffolds with native ECM ultrastructure and composition while removing all cells and genetic material present in the native tissue. Several decellularization protocols have been described for nearly all tissues and organs in the body, and include physical, chemical, and/or enzymatic approaches. Discussing the effects of these agents is beyond the scope of this review (reviewed in refs. ^165,166^); however, it is important to highlight that the choice of optimal protocol for tissue decellularization depends on many factors, including tissue source, cellularity, composition, density, thickness, and donor age, and these overall impact the efficiency of the decellularization process and the resultant ECM composition.

Cardiac tissue or whole heart decellularization is typically accomplished by using ionic and nonionic detergents. Decellularization of a whole rat heart through perfusion^167^ and of thin slices of human myocardial tissue^168^ using sodium dodecyl sulfate (SDS) resulted in lower DNA content and maintenance of native ECM structure, when compared with approaches using other chemicals. Typically, decellularizing human cardiac tissue^114^ requires a higher SDS exposure time than animal-derived cardiac tissue^169,170^, and the reasons for this can be manyfold, such as higher collagen and lipidic content, a stiffer ECM, or donor age. Because of this, it is becoming clear that interspecies differences may hinder the use of decellularization protocols optimized in animals for human tissue. The most immediate application of decellularized myocardial sheets (~200–400 µm thickness^113,114^), muscle strips (15 mm length, 2.5 mm diameter^114^), or whole heart^114^ is scaffold recellularization. Even though myocardial sheets and strips were successfully recellularized with CMs, recellularization of a human heart is an unmet goal and the best attempt so far has been an ~500 million CM intramyocardial injection to an ~5 cm^3^ volume of an acellular heart scaffold^114^, which is way below the billions of CMs necessary to repopulate this organ. Nevertheless, isolated ECM can be broken down, solubilized with pepsin, and then assembled into a nanofibrous hydrogel^171^, inevitably destroying the structural and mechanical architecture of the ECM scaffold. Interestingly, decellularized human cardiac ECM requires additional steps of lipid removal before hydrogel formation^172^. Soluble ECM in 3D settings promoted CM maturation^112^, but has a wider application for scaffold functionalization and has shown versatility to be implemented in advanced manufacturing techniques^169,173,174^. Despite its promising applications, decellularized human cardiac ECM is scarce due to donor organ shortage, and therefore research often relies on off-the-shelf animal-derived matrices.

### Microfabrication

Advances in microfabrication have allowed detailed engineering of material features in order to resemble tissue in vivo conditions. In particular, photolithography enables the precise transfer of geometrical shapes from a mask onto a substrate to obtain defined topographies for anisotropic tissue organization, while with soft lithography, substrates can be designed to include microchannels and complex microfluidic networks that are useful to recreate tissue vasculature. However, manufacturing such platforms can be time consuming, requires specialized equipment and facilities, and production may be costly.

The simpler systems use microfabrication to pattern material substrates, so as to ease CM spreading and sarcomere axial alignment. For instance, one of the initial attempts used polydimethylsiloxane (PDMS) thin films that contained an array of alternating 20-µm-wide lines, and these grooves substantially improved CM alignment and showed compliance during the cardiac cycle^110^. Currently, more sophisticated patterned culture platforms have been established, which synergize well with biochemical cues. In fact, circular chambers (ranging from 200 to 600 µm diameter), obtained through plasma etching of polyethylene glycol substrates, allowed geometrical confinement of hiPSC colonies and mediated morphogenesis of cardiac microchambers during differentiation^122^. Patterned culture chambers can also be useful to support hydrogel-based cardiac tissue formation (~3–4 mm length) and its long-term maturation (e.g., Biowire^TM^ II^175^). Nonetheless, microfabrication methodologies can be harnessed to create complex tissues similar to the native myocardium. Pioneering work used laser microablation of poly(glycerol sebacate) membranes to produce an accordion-like honeycomb scaffold, which was designed by overlapping two 200 µm × 200 µm squares at a 45° angle^176^. The unconventional design was purposely conceived to closely resemble the heart anisotropy, and proved successful in guiding CM alignment and improving their mechano-microenvironment. In another study, a porous photomask (2.5 cm × 2.5 cm) was crafted based on cardiac fiber orientation of a specific epicardial plane retrieved from a 3D reconstruction of the human ventricle^125^. This mask was used to mold a PDMS substrate that allowed a patch-like cardiac tissue formation in a developmentally mimetic fashion. More contemporary improvements have advanced even further cardiac patch design by using conductive biomaterials with built-in nanoelectronics^177,178^ (~20 mm × 5 mm), which are practical for online electrophysiological recording and electromechanical pacing.

Simple fabrication technologies, such as hydrogels, are unable to integrate organized vasculature within engineered tissue; however, microfabricated cardiac tissues can be assembled with perfusable and highly branched endothelialized channels^179,180^ by relying on simple soft lithography approaches. In particular, the AngioChip device (5 mm × 3.1 mm × 150–300 µm), a biodegradable elastomer assembled with layer-by-layer 3D stamping, reported unprecedented functional vascularization of cardiac tissues, allowing perfusion through an open lumen, capillary outgrowth, enhanced permeability, structural integrity, and matrix remodeling^180^.

Despite the lack of standardized culture modes across these systems, remarkable tissue-level complexity can be achieved, as well as adequate tissue function for high-throughput analysis^175^.

### Electrospinning

Electrospinning is a versatile method to fabricate ECM-like scaffolds as braided, woven, or knitted fibrous networks, from both naturally derived and synthetic materials. In solution electrospinning, the polymer is gravitationally forced to pass through an electrostatic field to generate a fibrous meshwork. Even though electrospun fibers frequently display inappropriate porosity compromising cell spreading and infiltration, certain parameters, like voltage and polymer flow rate, can be readily adjusted to fabricate the desired fibrous architecture. We have used solution electrospinning to produce poly(ε-caprolactone) (PCL) electrospun scaffolds (~2 cm × 2.5 cm × 229 nm), and the resulting fibers displayed an appropriate architecture that allowed CM alignment^181^. Furthermore, coating the scaffold with piezoelectric microfibers provided suitable electrical stimuli to the cells.

Over the past few years, there have been proposed other electrospinning-based techniques, which aimed at improving certain drawbacks of the traditional method, such as dependency on the polymer’s solution conductivity, slow deposition rates, and imprecise fabrication of 3D structures. Producing nano- to sub-micrometer scale fibers with a fast rate can be achieved using a high-speed rotating reservoir that propels centrifugal polymer deposition in a cylindrical collector^182^. The resulting polyester scaffold (25 mm diameter) was shown to promote CM alignment. A more recent approach used an ellipsoidal mandrel to collect electrospun PCL gelatin-coated nanofibers^127^. The ventricle-inspired structure (truncated ellipsoid; 4.5 mm diameter, 9 mm height) promoted CM laminar alignment, even though the ejection fractions and contractile work were ~50–250 and ~10^8^ smaller than the physiologic values for a human ventricle, respectively. Another promising methodology is melt electrospinning (also known as melt electrowriting), which allows a more controlled scaffold fabrication by precise collector or nozzle positioning, similar to 3D printing. Despite being unsuitable for cell encapsulation purposes, a relatively recent study demonstrated that a foldable PCL melt electrospun hexagonal-shaped scaffold (8 mm diameter, 300 µm thick) contributed to CM alignment and maturation^183^. Future improvements to these techniques or integrating existing expertise in multistep biofabrication approaches could allow their widespread use for cardiac TE.

### Bioprinting

Bioprinting is an emerging biofabrication method based on precise layer-by-layer deposition of biomaterials, biochemicals, and living cells, collectively named bioinks^184^. High-fidelity tissue and organ surrogates can be generated with the aid of medical imaging modalities, namely computed tomography and magnetic resonance imaging.

Synthetic materials were amongst the first to be printed, but now research focuses on using naturally derived materials, such as ECM hydrogels derived from decellularized tissue. In particular, cardiac-specific ECM-derived bioinks, when pressure extruded through a printing nozzle, have successfully generated grid-type structures in the form of rectangles^169^ (~5 mm × 5 mm × 1 mm) or circles^173^ (10 mm diameter, 0.6 mm thick), such that it resembles a patch. Extrusion bioprinting is one of the most popular methods to directly print cell-laden hydrogel-based cardiac tissue in an organized and reproducible fashion, besides allowing printing with high cell densities^184^, which is a necessary requirement to print more complex structures. Aside from the above-mentioned simple tissue constructs, extrusion bioprinting can be used to create more complex tissue models, but freeform printing of such structures requires a supporting sacrificial material. A novel method termed “FRESH” (i.e., Freeform Reversible Embedding of Suspended Hydrogels) achieved extrusion-based freeform printing by embedding the bioink in a thermally reversible supportive hydrogel during printing^185,186^, and this allowed fabrication of high-resolution 3D anisotropic structures, including a beating ventricle (truncated ellipsoid; 5.7 mm diameter, 8 mm height) and an acellular neonatal-sized heart analog (37 mm diameter, 55 mm height)^186^. Another study used a similar approach, but with an enzymatic or chemical reversible supportive hydrogel, and was able to print a heart-shaped model (14 mm diameter, 20 mm height) using an omental ECM biomaterial in combination with CMs and endothelial cells^174^. Further improvements are needed to create a functional tissue since the cells were reported to be arranged as nonaligned aggregates after 1 day in culture.

Another advantage of bioprinting is the possibility of creating tissues with inherent perfusable vasculature^187–189^. Direct extrusion-based bioprinting was used to manufacture one of the first vascularized cardiac tissues, in which the resulting endothelialized grid-type structure (~5.5 mm × 3.5 mm × 0.75 mm) was seeded with CMs and perfused with a microfluidic device^187^. Bioprinting a vascular network greatly improves the thickness these tissues can achieve without becoming necrotic (>1 cm^188^), but they often lack physiologically relevant cell numbers (~1 × 10^8^ cell/mL). A new innovative method termed “SWIFT” (i.e., Sacrificial Writing Into Functional Tissue) relies on assembling up to 400,000 spheroids/organoids as a tissue matrix and then extrudes a sacrificial ink throughout the compact granular tissue, which can later be evacuated and the resulting lumen perfused^190^, despite lacking robustly endothelialized channels. This clever technique allowed the assembly of a cardiac tissue with 240,000 million cells (6 mm top width, 4.2 mm bottom width, 12 mm height, 4.2 mm depth) that fuse and beat synchronously over 7 days in culture. Stereolithography, a higher resolution bioprinting methodology that uses a light projector to solidify layer-by-layer the bioink, has recently been used to create entangled vascular networks to perfuse an alveolar sac model^189^. Due to its exceptional level of functionality, translating this approach to cardiac TE could also be worthwhile.

It is important to take into account that additive printing with a high level of detail requires a slow printing velocity, affecting the scalability of current methods. To overcome this limitation, a recent study demonstrated that tissue can be printed within seconds with high fidelity by irradiating multiple light patterns representing projections of the object to be printed onto a photopolymer^191^. Despite being a promising technology, its potential use for cardiac TE still needs to be evaluated.

## Unlocking cardiac tissue engineering for precision medicine

**Fig. 4:**
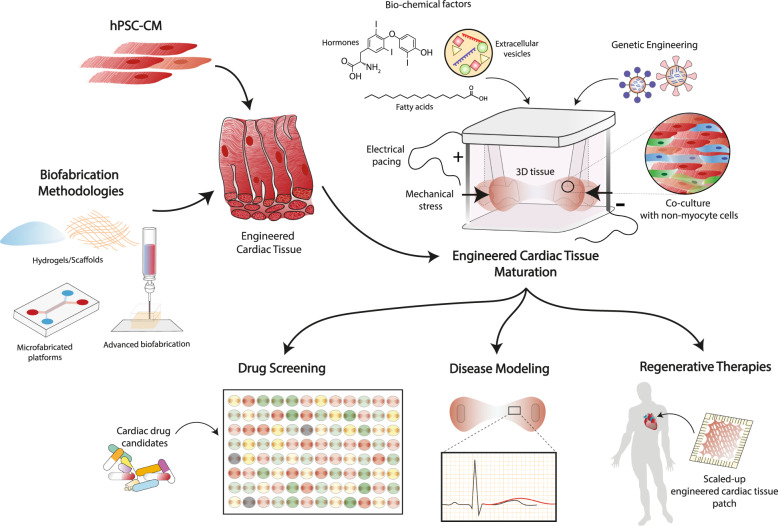
Streamlining engineered cardiac tissues for precision medicine applications. Tissue engineering expertise enables the generation of physiologically relevant cardiac tissues grown from human pluripotent stem cell-derived cardiomyocytes (hPSC-CMs). Designing a proper maturation strategy will allow the maturation of hPSC-derived cardiac tissues to an adult-like phenotype, which can later on be used for patient-specific drug screening, disease modeling, and regenerative therapies.

The advances in hPSC biology together with the development of novel materials and biofabrication methods have enabled the production of patient-specific cardiac tissues, which in turn opens up the possibility of fulfilling the promise of personalized medicine (Fig. 4). This section includes a brief overview of the current state-of-the-art applications of cardiac TE for drug discovery, disease modeling, and regenerative therapies.

### Cardiac tissue engineering for drug discovery and disease modeling

It is well known that cardiovascular safety liabilities remain a major cause of drug attrition, resulting in early termination of pipeline candidates or withdrawal of marketed drugs^192^. Commonly used platforms to assess safety include small animal models, primarily mice, and ion-channel overexpressing cell lines^193,194^, which fail to truly predict human drug response effects (proarrhythmic, cardiotonic, and cardiotoxic) due to differences in cardiovascular physiopathology. Thus, cardiac TE-based solutions, such as spheroids^120,195^, microtissues^63,124^, and “heart-on-a-chip”/microfluidic devices^175,196,197^, have been emerging as advanced culture systems, capable of improving the physiologic relevance over traditional 2D in vitro culture platforms. In these systems, hPSC-CMs have shown higher predictive insight regarding drug mechanistic (e.g., inotropic response^101,175,198^ and ECC blockage^101,175^) and toxic (e.g., loss of contractile function^198^ and apoptosis^195^) effects. Nonetheless, additional efforts are required to improve the reliability of these systems. For instance, a recent study demonstrated that linsitinib, a promising drug candidate to treat Ewing sarcoma, significantly reduced tumor viability and cardiac function when both of these engineered tissues were cultured separately under perfusion in an organ-on-a-chip system. However, when they were cultured in an integrated way in the same platform, linsitinib showed poor tumor response and less cardiotoxicity, which was in agreement with the unfortunate clinical trial outcome of this drug^199^. Testing the targeted or off-targeted effect of new cardiovascular drugs will require advancing the physiological relevance in which cardiac engineered tissues are cultured. In addition, the complexity of engineered cardiac tissues is still incompatible with a rapid screen of large libraries of compounds, despite their smaller size in relation to a whole tissue/organ. Although some platforms have shown the ability to be scaled-down^124,200,201^, high-throughput culture modes and reproducible assessment of tissue functionality are still lacking^202^.

Expediting drug development will further require reliable models for a better understanding of disease mechanisms that guide target drug validation/discovery. In this context, hPSCs, as stated above, have emerged as attractive tools and a preferred source for obtaining cells for in vitro models, since these cells can be derived from patients using minimally invasive procedures, and preserve the genetic and phenotypic characteristics of the donors, including pathological traits responsible for disease onset and progression^203,204^. Several studies have been using 2D hiPSC-CM systems to investigate a plethora of genetic mutations associated with genetic heart diseases, including the main inherited cardiomyopathies (i.e., familial hypertrophic cardiomyopathy^205^, dilated cardiomyopathy^203,206^, arrhythmogenic cardiomyopathy^207^, and left ventricular noncompaction cardiomyopathy^208^) and channelopathies (e.g., type-2 long-QT syndrome^209^ and Timothy syndrome^210^). Others are modeling diseases without a known disease-causing genetic variant, such as idiosyncratic drug-induced cardiotoxicity^204,211,212^. In parallel with the use of patient-derived hPSCs, recent advances in targeted gene editing using the CRISPR-Cas9 systems have been performed on hPSCs^213–215^. Employing this technology, any wild-type cell can be altered to harbor a specific mutation, and a disease-causing mutation can be corrected in patient-derived cells, generating an isogenic control cell line. To the best of our knowledge, there are 13 completed or active clinical studies (www.clinicaltrials.gov) with the goal of generating patient-specific CMs to study the molecular mechanisms involved in several cardiac diseases, such as genetic and hypertrophic cardiomyopathies, inherited arrhythmias and valvulopathies, and cardiac fibrosis, among others (Table 1).

**Table 1:** Human clinical studies for the generation of disease and/or patient-specific models of cardiac diseases (Trial Identifier: www.clinicaltrials.gov).

Study title	Condition(s)	Study type	Trial identifier
Individualized early risk assessment for heart diseases (IndivuHeart)	Hypertrophic cardiomyopathy; dilated cardiomyopathy	Observational	NCT02417311
Investigating hereditary cardiac disease by reprogramming skin cells to heart muscle (CLUE)	Electrophysiology of iPSC-CM	Observational	NCT01865981
Modeling and pharmacological targeting of genetic cardiomyopathy in children via iPSC-CM (DMDstem)	Familial cardiomyopathy	Interventional	NCT03696628
Molecular mechanism identification in inherited arrhythmias and valvulopathies from iPSC (Diag-iPS)	Inherited arrhythmias and valvulopathies	Interventional	NCT01734356
Evaluating cardiovascular phenotypes using iPSC	Coronary artery disease	Observational	NCT01517425
Generation of Marfan syndrome and Fontan cardiovascular models using patient-specific iPSC	Marfan’s syndrome	Observational	NCT02815072
Translational approaches to septic cardiomyopathy (TASC01)	Cardiomyopathies, sepsis, septic shock	Observational	NCT03252613
CIQTP prolongation: role and mechanism in sudden cardiac death (IQARE-SCD)	Sudden cardiac death	Observational	NCT03387072
Derivation of iPSC to heritable cardiac arrhythmias	Inherited cardiac arrhythmias; long QT syndrome; Brugada syndrome; catecholaminergic polymorphic ventricular tachycardia; early repolarization syndrome; arrhythmogenic cardiomyopathy; hypertrophic cardiomyopathy; dilated cardiomyopathy; muscular dystrophies (Duchenne, Becker, myotonic dystrophy)	Observational	NCT02413450
Early MRI detection of myocardial deterioration as a preventive, disease staging, and prognostic biomarker in insulin resistance	Cardiomyopathies; insulin resistance; non-ischemic cardiomyopathy; cardiac fibrosis; diabetes	Observational	NCT03509441
Characterization of patients with uncommon presentations and/or uncommon diseases associated with the cardiovascular system	Cardiomyopathy; Li-Fraumeni syndrome; Parkinson’s disease; atherosclerosis; cardiovascular capacity	Observational	NCT01143454
UTHealth Turner syndrome research registry	Turner syndrome	Observational	NCT03185702

*iPSC* induced pluripotent stem cells, *iPSC-CM* induced pluripotent stem cells derived cardiomyocytes, *MRI* magnetic resonance imaging.

Notwithstanding, the use of engineered cardiac tissues as disease models is a relatively new concept. hPSC-CM tissue platforms harboring genetic mutations characteristic of hypertrophic cardiomyopathy^216^, dilated cardiomyopathy^217^, and mitochondrial cardiomyopathy of Barth syndrome^218^ have reported abnormalities in cardiac function congruent with a pathological phenotype. More recently, chamber-specific arrhythmia was demonstrated in a ring-shaped cardiac tissue containing both ventricular and atrial hPSC-CMs^219^. Manipulating engineered tissue microenvironment to induce pathological changes, such as CM remodeling of myocardium failure^220^, excessive ECM deposition of cardiac fibrosis^221^, and thrombus formation^222^, could similarly provide invaluable insights into mechanistic studies or predict patient-specific disease progression. Even though promising, disease-carrying hPSC-CMs still present an immature phenotype, thus possibly confounding identified disease hallmarks and identified mechanisms. For instance, genetically engineered hPSC-CM microtissues carrying mutations that truncate the sarcomere protein titin, a known common cause of dilated cardiomyopathy, exhibited reduced contractility and decreased sarcomere length, among other abnormalities^217^, none of which were detected in adult samples^223^. As a matter of fact, cardiac tissue structural maturity imposed by intensive electrical training was necessary to model pathological ventricular hypertrophy^101,175^. Even with their current limitations, the identified pathomechanisms in diseased engineered cardiac tissues can, in some cases, be translated to patient care in a personalized way. Recent research found out diltiazem, an l-type calcium channel blocker, was effective to correct disease-specific cardiac abnormalities in patient-derived engineered muscle strips with a rare form of hypertrophic cardiomyopathy. Diltiazem was further translated into patient care and ameliorated the prolonged QTc interval after 1 month of treatment^224^. These encouraging results give hope that emerging TE platforms could be extremely useful to reverse the growing burden associated with cardiovascular disorders.

### Cardiac tissue engineering for heart regenerative therapy

Regenerative cardiac cell therapy broadly aims to achieve two complementary goals: (i) the direct cell replacement of the injured myocardium with contractile CMs and (ii) paracrine modulation of endogenous repair processes, such as angiogenesis, inflammation, apoptosis, and fibrosis, with both contractile and noncontractile cells^14,225,226^. To the best of our knowledge, there are over 40 completed clinical trials exploring the therapeutic potential of cell-based therapies for cardiac regeneration and repair (www.clinicaltrials.gov). Pioneer studies tested non-cardiomyogenic cell populations that can easily be prepared for clinical applications, namely bone marrow-derived mononuclear cells (BM-MNCs) or MSCs, which in spite of their limited regenerative potential, may stimulate endogenous regenerative responses^225,227^. Second-generation cell candidates require a more refined isolation process, and ex vivo amplification procedures, but have higher regenerative potential, such as cardiac-derived progenitor cells and hPSC-CMs^225,227^. However, the clinical outcome of most of these studies remains insufficient, with evidence suggesting the major common obstacles to be the low engraftment and survival rate of transplanted cells in the host tissue, and the scarcity of endogenous cells with repair capacity^225,228–230^.

Cardiac TE has proven to be an effective method to improve the delivery, engraftment, and differentiation of stem cells in injured hearts, and therefore has gained much attention in recent years. Cardiac tissue surrogates developed to date are typically engineered cardiac muscle strips^231–233^, epicardial patches^126,234–236^, or cardiogenic scaffolds^237^, even though other strategies can be employed^238–243^, such as acellular hydrogels to improve infarct mechanical integrity^241–243^. A summary of relevant cardiac TE preclinical studies in animal models is highlighted in Table 2. When transplanted into infarcted or defected animal hearts, hPSC-CM-based tissues led to longer-term cell retentions^231^, progressively matured in vivo^231^, (imperfectly) electrically coupled with the host tissue^233^, reversed infarction-associated changes (e.g., ameliorated cardiac remodeling by decreasing the amount of fibrosis^241–243^, enhanced vascularization^126,233,242^), and overall improved left ventricular function^231,240,243^. Recent advances have allowed rapid fabrication of clinically relevant sized human cardiac tissues able to maintain functionality preimplantation^126,234^, thus being suitable for large animal preclinical studies and future clinical applications.

**Table 2:** Selected in vivo preclinical studies on cardiac tissue engineering.

Tissue engineering strategy	Specific tissue engineering approach	Cell source	Animal model	Disease model	Major achievements	Ref.
Engineered heart tissue	CM and collagen type I macroscale ring EHT	hESC-CM	Rat	MI IR 60 min	*Cardiac function recovery*: no significant changes in LVEDV and LVESV at 4 weeks; progressive improvement in LVEF at 4 weeks *Assessment methods*: echocardiography and MRI	^231^
Engineered heart tissue	Fibrin EHT	hiPSC-CM, -EC	Guinea pig	Cryo-injury	*Cardiac function recovery*: absence of pro-arrhythmogenic effects at day 28 *Assessment methods*: echocardiography	^232^
Engineered heart tissue	Fibrin EHT	hiPSC-CM, -EC	Guinea pig	Cryo-injury	*Cardiac function recovery*: improved LV function at day 28; EHT vascularization and electrical coupling with host heart tissue *Assessment methods*: ecochardiography	^233^
Cardiac patch	﻿3D-printed patch composed of hyaluronic acid/gelatin-based matrix.	﻿Human CPC	Mouse	MI	*Cardiac function recovery*: reduction in LVEDV and LVESV at 4 weeks; reduction of infarct fibrosis Assessment methods: MRI and histology	^237^
Cardiac patch	Fibrin patch with nylon frame	hiPSC-CM	Rat	N/A	*Cardiac function recovery*: patches failed to electrically couple with the recipient’s hearts; patch vascularization by host vessels; no immune rejection *Assessment methods*: electromechanical optical dual mapping, dorsal window chamber assay, and histology	^126^
Cardiac patch	Cardiac muscle patch	hiPSC-CM, -EC, and -SMCs	Pig	MI IR 60 min	*Cardiac function recovery*: LVEF and LVEDV improvements at 4 weeks; no spontaneous arrhythmias were detected 2 weeks post-acute MI phase *Assessment methods*: MRI and electrocardiography	^235^
Cardiac patch	3D fibrin patch loaded with insulin growth factor-encapsulated microspheres	hiPSC-CM, -EC, and -SMC	Pig	MI	*Cardiac function recovery*: improved LVEF at 4 weeks; reduction in LV wall stress; reduction in infarct size *Assessment methods*: ecochardiography and histology	^234^
Cardiac patch	Conductive patch composed of chitosan, phytic acid, and aniline	Acellular	Rat	MI	*Cardiac function recovery*: improvements in LVEF and LVFS at 2 weeks; absence of pro-arrhythmogenic effects *Assessment methods*: echocardiography	^236^
Cardiac patch	Viscoelastic starch patch designed by finite-element simulation	Acellular	Rat	MI	*Cardiac function recovery*: decreased LVIDD and LVIDS at 4 weeks; improved LVEF and LVFS at 4 weeks; reduction in infarct size; reduction of myocardial hypertrophy *Assessment methods*: echocardiography, histology, and immunofluorescence microscopy	^243^
Microspheres	Gelatin MSs	CPC and CPC + MS	Mouse	MI	*Cardiac function recovery*: improved LVEF at day 28; reduction in LVESV and LVEDV *Assessment methods*: MRI	^238^
Nanofibers	Poly(d,l-lactic-*co*-glycolic acid) polymer nanofibers	hiPSC-CM	Rat	MI	*Cardiac function recovery*: improvements in LVEF, LVFS, and LVESD at 4 weeks; no immune rejection *Assessment methods*: echocardiography and histology	^239^
Cell sheets	Cell sheet	hiPSC-CM	Pig	MI	*Cardiac function recovery*: improvements in LVEF, LVEDV, and LVESV at 4 and 8 weeks *Assessment methods*: echocardiography and cardiac MSCT	^240^
Biomaterials	Injectable alginate hydrogel	Acellular	Rat	MI	*Cardiac function recovery*: LVFS improvement at 8 weeks after injection, either in recent or old infarcts *Assessment methods*: echocardiography	^241^
Biomaterials	Solubilized porcine myocardial ECM injectable hydrogel	Acellular	Pig	MI	*Cardiac function recovery*: LVEF, LVEDV, and LVESV improvements at 12 weeks; reduced infarct size at 3 weeks *Assessment methods*: echocardiography and NOGA	^242^

*CM* cardiomyocytes, *CPC* cardiac progenitor cell, *EC* endothelial cells, *ECM* extracellular matrix, *hESC-CM* human embryonic stem cells derived cardiomyocytes, *hiPSC* human induced pluripotent stem cells, *hiPSC-CM* human induced pluripotent stem cell-derived cardiomyocytes, *IR* ischemia–reperfusion, *LV* left ventricular, *LVEDV* left ventricular end-diastolic volume, *LVEF* left ventricular ejection fraction, *LVESD* left ventricular end-systolic dimension, *LVESV* left ventricular end-systolic volume, *LVFS* left ventricular fractional shortening, *LVIDD* left ventricular internal diastolic diameter, *LVIDS* left ventricular internal systolic diameter, *MI* myocardial infarction, *MRI* magnetic resonance imaging, *MS* microspheres, *MSCT* multislice computer tomography, *SMC* smooth muscle cells.

Among relevant risks that have to be taken into account in these therapies are the development of arrhythmias^244,245^, uncontrolled proliferation, and unwanted differentiation of cells that may lead to tumor formation in the case of hPSC-based therapies^245–248^, or to calcification of the host myocardium by MSCs or BM-MNCs^247,249^. Another potential issue is the development of an adverse immune response against the cells and materials employed in the constructs^250^. Designing tissues with a more controlled cell composition, defined architectural structure and developing strategies favoring muscular maturation before implantation could further improve their regenerative capability.

Despite showing promising outcomes in preclinical settings, the potential use of cardiac TE-based therapies at a clinical level is still in its infancy. Preliminary data from first-in-man clinical trials are providing evidence that cardiac TE-based therapies are generally safe^251,252^, but do not provide relevant functional benefits. We are aware of five completed and two active phase 1/2 studies mainly aiming at evaluating the safety and feasibility of cardiac TE therapeutic interventions, but also collecting preliminary efficacy data (Table 3).

**Table 3:** Human clinical trial for cardiac tissue engineering (Trial Identifier: www.clinicaltrials.gov).

Study title	Condition	Intervention material	Phase and status	Trial identifier	Refs.
IK-5001 for the Prevention of Remodeling of the Ventricle and Congestive Heart Failure After Acute Myocardial Infarction (PRESERVATION-1)	Acute MI; congestive HF; ST-elevation MI	IK-5001 (sodium alginate and calcium gluconate hydrogel)	Phase 1 Completed	NCT01226563	^256–258^
A randomized, controlled study to evaluate Algisyl-LVR™ as a method of left ventricular Augmentation for Heart Failure (AUGMENT-HF)	HF; dilated cardiomyopathy	Algisyl (calcium alginate hydrogel)	Phase 2 Completed	NCT01311791	^259–261^
Epicardial infarct repair using CorMatrix^®^-ECM: clinical feasibility study	Acute coronary syndrome; HF	CorMatrix-ECM	Phase 1 Completed	NCT02887768	^252,254^
A study of VentriGel in post-MI patients	MI; HF; left ventricular remodeling	Ventrigel (ECM hydrogel)	Phase 1 Completed	NCT02305602	^255^
Transplantation of hESC-derived progenitors in severe heart failure	Ischemic heart disease	hESC-derived CD15^+^Isl-1^+^ progenitors embedded into a fibrin patch	Phase 1 Completed	NCT02057900	^251,262^
Randomized study of coronary revascularization surgery with an injection of WJ-MSCs and placement of an epicardial patch	Cardiovascular diseases; HF; coronary artery disease	ECM patch with WJ-MSC	Phase 1 and 2 Active, not yet recruiting	NCT04011059	–
Safety and efficacy of iPSC-derived engineered human myocardium as Biological Ventricular Assist Tissue in Terminal Heart Failure (BioVAT-HF)	HF	Engineered heart muscle	Phase 1 and 2 Active, recruiting	NCT04396899	–

*ECM* extracellular matrix, *hESC* human embryonic stem cells, *HF* heart failure, *MI* myocardial infarction, *WJ-MSC* Wharton’s jelly-derived mesenchymal stem cells.

The first studies being conducted are focused on the hypothesis that biomaterials could act as an internal wall support to increase infarct wall thickness, thereby improving cardiac function. Results from PRESERVATION I and AUGMENT-HF trials were, however, disappointing, with minor improvements in symptoms and clinical status for patients with advanced heart failure (HF), but no significant changes in left ventricle (LV) remodeling. Aiming to provide not only mechanical support for the prevention of infarct expansion but also to restore ECM homeostasis and architecture, other strategies relied on the epicardial or myocardial application of ECM patches or hydrogels. In particular, CorMatrix^®^-ECM patches, derived from porcine small intestinal submucosa (SIS-ECM), were applied on the epicardium of patients undergoing coronary artery bypass grafting, after an acute ischemic event. Porcine SIS-ECM has been used for over 20 years in a range of cardiovascular applications, but of all the commercially available SIS-ECM products, CorMatrix^®^ is the only medical device with clearance, even though there are few reports regarding its use for myocardial regeneration after acute myocardial infarction in humans^253^. No results for this study have been published so far; however, based on preliminary data obtained in rodent and porcine models, the patches are expected to not only prevent infarct expansion and LV dilation but also restore ECM homeostasis and architecture^252,254^. Another recently completed study evaluated the effect of VentriGel, a myocardium-specific ECM hydrogel, on patients in a 60- to 3-year window since their first acute ST-elevation myocardial infarction. Using a minimally invasive procedure, the gel was injected using a NOGA-MyoStar catheter, allowing for an intramyocardial delivery of the therapeutic gel on mapped infarcted areas. Overall, VentriGel showed encouraging safety results, with no deaths or patients who discontinued from the study, nor adverse events reported as a direct consequence of the treatment^255^. Although efficacy was not a primary outcome, there were suggestions of improvements, including in the 6-min walk test distance and decreases in New York Heart Association functional classification, across the entire cohort of patients^255^. Improvements in LV remodeling data, viable mass, and ventricular natriuretic peptide levels were mainly observed in patients who had a myocardial infarction >12 months before treatment^255^.

A distinctive approach has been the delivery of clinical-grade hESC-derived cardiovascular progenitor cells in fibrin patches for the treatment of severe ischemic left ventricular dysfunction^251^. Compared with biomaterial-only methods, cell patches showed equal safety profiles, with no complications that could be specifically ascribed to the cellular graft, namely, no detection of teratomas that could be developed from residual pluripotent cells, no occurrence of arrhythmias, and no major alloimmunization events. Still, following a similar trend as previous strategies, no astonishing results were obtained in terms of efficacy, although all patients were symptomatically improved. A newly posted study aims at determining the safety of Wharton’s jelly-derived MSCs from human umbilical cord seeded on ECM patches, and also test its efficacy to promote neomyogenesis in patients with previous myocardial infarction. Another active and recently recruiting promising clinical trial will evaluate the efficacy of an engineered heart muscle (EHM) to sustainably remuscularize and improve myocardial performance in patients with advanced HF with reduced ejection fraction (≤35%). The EHM, a construct generated from hiPSC-CMs and stromal cells in a bovine collagen type I hydrogel, will be the first of its kind to be tested in humans.

Despite the marginally positive outcome of all these clinical trials, this does not necessarily imply a lack of therapeutic value and may result instead from an inadequate choice of biomaterial and/or cell type or inaccuracies in dosing and/or delivery. A better understanding underlying the mechanism of action of these advanced medicinal products may contribute to further improvements down the road.

## Conclusions

To date, inducing maturation of hPSC-CMs is a challenge in the field, limiting their use in clinical settings. Considerable progress has been achieved by continuously seeking innovative ways to improve hPSC-CM maturation state, and, currently, it is already possible to obtain cardiac tissue with adult-like gene signatures, remarkable ultrastructural organization, functional contractile machinery, and improved electrophysiology. Even though in vitro maturation may not follow the in vivo paradigm, identifying the spatio-temporal mechanisms dictating tissue development and designing a combinatorial strategy capable of addressing such complexity might be required to engineer fully matured biomimetic cardiac tissues. Given the interdisciplinarity of TE, from stem cell biology to stem cell bioprocessing, material science, and advanced biofabrication, taking advantage of this knowledge would be beneficial to grow physiologically relevant cardiac tissues in vitro. Recreating cardiac tissue complexity is still challenging, limiting the size of the engineered tissue possible to be obtained, and thus redirecting their application towards other areas rather than the clinical transplantation arena. Benchmarked “on-a-chip” engineered cardiac tissues, although promising, still need to show standardization and high-throughput compatibility to contribute to more reliable results than commonly used platforms, as well as reduction of costs to expedite drug development. Successful attempts at scaling up engineered cardiac tissues have made it possible to investigate their regenerative efficacy in large animal studies, and the results of pioneering TE-based clinical trials are eagerly awaited. While there are still few clinical trials, these have been an important step in the ability to engineer artificial constructs that mimic some of the important compositional, architectural, and functional properties of human cardiac tissues. Even if it is not unrealistic to foresee a translation of engineered cardiac tissues for in vitro clinical studies, their potential to remuscularize failing hearts and integrate the host tissue without adverse effects, despite a better retention, is still uncertain, and the causes associated with mild improvements unknown. Nonetheless, advances in the field of cardiac TE have undoubtedly imposed a new paradigm in cardiovascular science, paving the way one day for restoring or even replacing damaged hearts.
